# Vagus Nerve Stimulation Induced Motor Map Plasticity Does Not Require Cortical Dopamine

**DOI:** 10.3389/fnins.2021.693140

**Published:** 2021-08-23

**Authors:** Jackson Brougher, Camilo A. Sanchez, Umaymah S. Aziz, Kiree F. Gove, Catherine A. Thorn

**Affiliations:** ^1^Department of Neuroscience, University of Texas at Dallas, Richardson, TX, United States; ^2^Department of Bioengineering, University of Texas at Dallas, Richardson, TX, United States

**Keywords:** neural plasticity, VNS, dopamine, motor cortex, 6-OHDA, motor learning

## Abstract

**Background:** Vagus nerve stimulation (VNS) paired with motor rehabilitation is an emerging therapeutic strategy to enhance functional recovery after neural injuries such as stroke. Training-paired VNS drives significant neuroplasticity within the motor cortex (M1), which is thought to underlie the therapeutic effects of VNS. Though the mechanisms are not fully understood, VNS-induced cortical plasticity is known to depend on intact signaling from multiple neuromodulatory nuclei that innervate M1. Cortical dopamine (DA) plays a key role in mediating M1 synaptic plasticity and is critical for motor skill acquisition, but whether cortical DA contributes to VNS efficacy has not been tested.

**Objective:** To determine the impact of cortical DA depletion on VNS-induced cortical plasticity.

**Methods:** Rats were trained on a skilled reaching lever press task prior to implantation of VNS electrodes and 6-hydroxydopamine (6-OHDA) mediated DA depletion in M1. Rats then underwent training-paired VNS treatment, followed by cortical motor mapping and lesion validation.

**Results:** In both intact and DA-depleted rats, VNS significantly increased the motor map representation of task-relevant proximal forelimb musculature and reduced task-irrelevant distal forelimb representations. VNS also significantly increased tyrosine hydroxylase (TH+) fiber density in intact M1, but this effect was not observed in lesioned hemispheres.

**Conclusion:** Our results reveal that though VNS likely upregulates catecholaminergic signaling in intact motor cortices, DA itself is not required for VNS-induced plasticity to occur. As DA is known to critically support M1 plasticity during skill acquisition, our findings suggest that VNS may engage a unique set of neuromodulatory signaling pathways to promote neocortical plasticity.

## Introduction

Preclinical studies suggest that vagus nerve stimulation (VNS) paired with rehabilitation training is a promising approach for enhancing motor recovery after neural injury (Khodaparast et al., 2013; Pruitt et al., 2016; Ganzer et al., 2018; Meyers et al., 2018). Training-paired VNS induces significant neuroplasticity within the motor cortex (Porter et al., 2012; Hulsey et al., 2016, 2019; Morrison et al., 2019; Tseng et al., 2020), which is thought to be critical for successful motor rehabilitation (Di Lazzaro et al., 2010; Pruitt et al., 2016; Bundy and Nudo, 2019; Meyers et al., 2019). While the precise mechanisms underlying VNS efficacy remain unclear, VNS-driven cortical plasticity is known to depend on the coordinated signaling of multiple neuromodulatory systems (Hays, 2016). Cortical depletion of noradrenergic, serotonergic, or cholinergic fibers blocks VNS-driven cortical reorganization (Hulsey et al., 2016, 2019), consistent with the known contributions of each of these neuromodulators to synaptic plasticity (Rasmusson, 2000; Gu, 2002; Lesch and Waider, 2012; Vitrac and Benoit-Marand, 2017). Dopamine (DA) is similarly recognized as a plasticity promoting neuromodulator within neocortical circuits (Hosp and Luft, 2013; Guo et al., 2015), but the necessity of dopaminergic signaling in VNS efficacy has not been previously tested (Guo et al., 2015).

Several lines of evidence suggest that DA could play a key role in VNS-driven cortical plasticity. VNS increases the firing rates of noradrenergic neurons in the locus coeruleus (LC) (Hulsey et al., 2017), which are known to activate dopaminergic neurons in the ventral tegmental area (VTA) (Mejias-Aponte, 2016; Park et al., 2017). VTA then sends dopaminergic projections throughout the forebrain, including to M1 (Lindvall et al., 1974; Hosp et al., 2011). Vagal signaling has recently been shown to enhance the activation of midbrain dopaminergic neurons and to increase the expression of behaviors known to depend on dopaminergic signaling (Han et al., 2018; Fernandes et al., 2020).

Cortical dopaminergic signaling plays a critical role in motor learning and M1 synaptic plasticity. Behaviorally, early skill acquisition is associated with increased VTA activation (Leemburg et al., 2018), and disruptions in cortical dopaminergic signaling have been shown to impair motor learning (Molina-Luna et al., 2009; Hosp et al., 2011; Rioult-Pedotti et al., 2015). Synaptically, DA receptor antagonism inhibits long-term potentiation in M1 (Molina-Luna et al., 2009; Rioult-Pedotti et al., 2015), and dendritic spine growth and pruning are differentially controlled by D1 and D2 receptor subtypes, respectively (Guo et al., 2015). Interestingly, after a task becomes well-learned, movement-related VTA activation is reduced (Leemburg et al., 2018), and cortical DA depletion no longer impacts motor performance (Molina-Luna et al., 2009; Hosp et al., 2011). Combined, these studies suggest that cortical DA is necessary for promoting the M1 plasticity that underlies new skill acquisition.

We hypothesized that DA may also be a key mediator of VNS-driven cortical plasticity, as it is during initial motor learning. To test this hypothesis, we trained rats on a skilled reaching lever press task prior to implantation of VNS electrodes and 6-OHDA mediated M1 DA depletion. Our findings indicate that while VNS treatment may increase cortical catecholaminergic innervation in intact M1, DA itself is not required for VNS-driven cortical plasticity to occur. These results raise the possibility that VNS efficacy during stroke rehabilitation may depend on a set of neuroplasticity-promoting mechanisms that are distinct from those that underlie initial motor skill acquisition.

## Materials and Methods

All procedures were approved by the University of Texas at Dallas Institutional Animal Care and Use Committee in accordance with the National Institutes of Health guide for the care and use of laboratory animals.

### Animal Subjects

Twenty-seven male Long-Evans rats (RRID:RGD_5508398) were included in the study, aged 9–12 weeks at study start. Rats were housed in a 12:12 h reverse light cycle room (lights on: 6:00 p.m.) with ad libitum access to water. All training was performed during their active cycle. To accustom the rats to handling, an experimenter held the animals near their home cages for 10–20 min daily for 3–5 days. Rats then received two 30-min habituation sessions, delivered on consecutive days, in which they were allowed to freely explore the training booths. During training, animals were food restricted to not less than 90% of their free feeding weight. Prior to surgery, rats were pair housed; after surgery, animals were housed singly.

### Behavioral Training and Experimental Design

**FIGURE 1:**
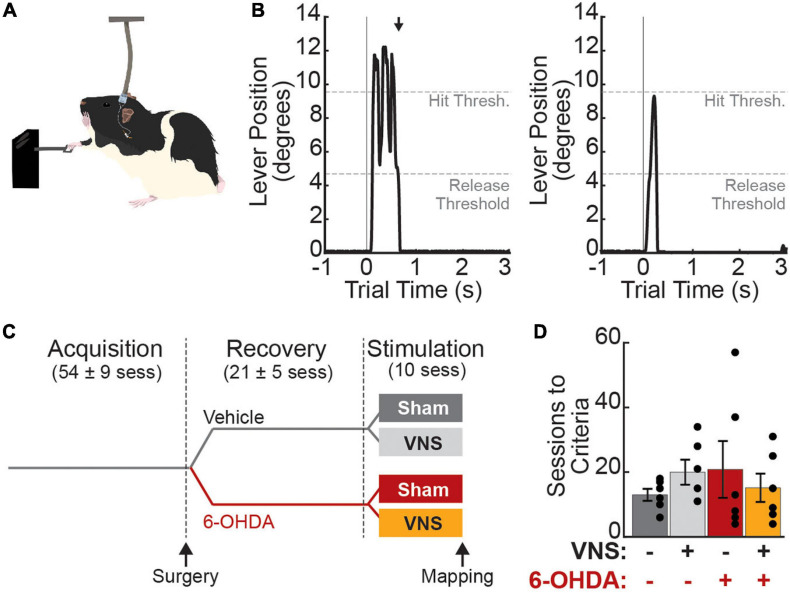
Experimental design. **(A)** Rats were trained to perform a skilled reaching lever press task with their right forelimb. During treatment sessions, vagus nerve (or sham) stimulation was paired with correct lever press performance. **(B)** Correct trials (“hit trials,” left) were those in which the lever deflection exceeded 9.5 degrees below horizontal (Hit Threshold) followed by a return to less than 4.75 degrees (Release Threshold) within 2 s. Black arrow denotes the time of reward delivery, which was coincident with detection of lever release. Miss trials (right) were those in which the hit threshold was not reached (shown) or in which the rat failed to release the lever within the 2 s trial window. **(C)** Rats were trained to criterial performance prior to VNS cuff implantation and 6-OHDA (or vehicle) infusions in left M1, i.e., contralateral to the trained limb. After behavioral recovery, rats received 10 stimulation sessions in which VNS or sham stimulation was paired with correct lever performance (*N* = 6 rats per treatment group). Values in parentheses are mean ± SEM for the number of sessions in each training epoch for all 24 rats in the main study. **(D)** Rats in all treatment groups required a similar number of sessions to first reach criterial task performance.

Rats were trained on a skilled-reaching lever press task as previously described (Tseng et al., 2020). Briefly, the task required the rats to reach 2 cm outside a MotoTrak training booth (Vulintus, Louisville, CO, United States) with their right forelimb to fully depress and release a lever within a 2 s window (Figures 1A,B) to receive a food reward (Bio-Serv, Flemington, NJ, United States). Lever position ranged from 0 (no deflection) to 13 (maximum deflection) degrees below horizontal and was continuously sampled at 100 Hz. A full lever press was detected when lever position exceeded 9.5 degrees, and lever release was subsequently detected when the lever position returned to less than 4.75 degrees from horizontal. During task acquisition, trials terminated and reward was delivered immediately upon detection of the correct lever press-and-release activity. Trials were terminated 2 s after trial initiation if a correct movement was not detected; no reward was delivered on these incorrect trials. Trials were followed by a 2 s time-out period and the next trial was then initiated upon detection of a lever deflection of greater than 0.5 degrees. Acquisition continued until criterial performance was reached (Figures 1C,D): at least 55 trials performed at over 65% correct performance in 8 of 10 consecutive training sessions, with the lever positioned 2 cm exterior to the booth.

Once criterial performance was reached, VNS cuff electrodes were implanted and intracortical 6-OHDA infusions were performed. At the time of surgery, rats were randomly assigned to receive either 6-OHDA or vehicle (veh; 0.9% NaCl saline) infusions. Animals were given a 2-week recovery period following surgery to allow lesions to stabilize. After recovery, rats were dynamically allocated to VNS or sham treatment groups (veh| sham: *n* = 6, veh| VNS: *n* = 6, 6-OHDA| sham: *n* = 6, 6-OHDA| VNS: *n* = 6) and returned to behavioral training until criterial performance was re-established. Rats then underwent 10 final training sessions in which VNS or sham stimulation was paired with correct lever press performance. Within 24 h of the last treatment-paired training session, somatotopic motor maps were obtained using intracortical microstimulation (ICMS).

In a small group of rats (*n* = 3), we tested the effects of pharmacological antagonism of DA receptors on VNS-driven cortical reorganization. Animals in this control group were trained identically to those in the main study. During surgery, these rats received implantation of a 22-gage infusion guide cannula into M1, along with chronically implanted VNS electrodes. After recovery, all rats in this group received 10 VNS-paired training sessions. Thirty minutes prior to each of these final treatment sessions, the D2 antagonist raclopride (10 μg/μL) and the D1 antagonist SCH 23390 (600 μg/μL) were co-infused into M1 (1 μL total volume, infused at 0.25 μL/min). All pharmacological agents were purchased from Tocris Biosciences (Minneapolis, MN, United States).

### VNS Cuff Implantation and 6-OHDA Administration

Vagus nerve stimulation cuff electrodes were implanted around the left cervical vagus nerve as previously described (Hulsey et al., 2016; Tseng et al., 2020). Briefly, custom peripheral nerve stimulating cuff electrodes containing two low-impedance platinum:iridium leads were assembled in-house according to published methods (Sanchez et al., 2020). Rats were anesthetized with ketamine/xylazine (80/10 mg/kg, i.p.), an incision was made approximately 1 cm from the midline on the left side of the neck, and the left vagus nerve was exposed via blunt dissection and placed inside a cuff electrode. Cuff leads were tunneled subcutaneously and exited through an incision made over the skull. Cuff validation was performed by stimulating the vagus nerve using a 10 s train of 0.8 mA, 100 μs biphasic pulses delivered at 30 Hz to elicit a brief cessation of breathing and drop in SpO2 consistent with the Hering–Breuer reflex (Paintal, 1973) prior to closure of the neck incision with sutures.

After electrode implantation, the rat was placed into a stereotaxic frame and a small craniotomy and durotomy were made over the forelimb region of left M1 (AP: +0.5 mm; ML: +2.5 mm), i.e., contralateral to the trained forelimb. A 26-gauge infusion needle (Hamilton, #7768-02) was lowered 1.0 mm below the pial surface, and 1 μL of 6-OHDA (6 μg/μL in saline) or saline vehicle was infused (0.1 μL/min). Sixty minutes prior to 6-OHDA infusion, the norepinephrine reuptake inhibitor desipramine (Tocris Biosciences, Minneapolis, MN, United States) was administered (20 mg/kg, i.p.) to preserve noradrenergic fibers. Rats receiving intracortical saline infusions received i.p. injections of an equivalent volume of sterile saline. Once cortical infusions were complete, VNS cuff electrode leads were connected to a headcap connector, which was then secured to the skull with four bone screws and dental cement. Rats were provided with Baytril (enrofloxacin, 0.5 mg/5 g) and Rimadyl (carprofen, 2 mg/5 g) tablets (Bio-Serv, Flemington, NJ, United States) for 3 days post-surgery.

For rats in the M1 DA antagonist group, no infusions were performed during surgery. Rather, a 22-gauge steel cannula (Guide 38172, Plastics1, Roanoke, VA, United States) was implanted in M1, using the same coordinates as above, to enable intracortical administration of raclopride and SCH 23390 prior to VNS treatment sessions.

### VNS Administration

Vagus nerve stimulation was administered according to protocols previously established to induce significant reorganization of the cortical motor map (Porter et al., 2012; Tseng et al., 2020). During training-paired VNS treatment sessions, stimulation was delivered immediately upon detection of a successful lever press. Stimulation parameters were identical to those used in prior studies (Porter et al., 2012; Tseng et al., 2020) and consisted of a 0.5 s train of 16 pulses (amplitude: 0.8 mA, pulse frequency: 30 Hz, and pulse width: 100 μs biphasic). Sham-treated rats were similarly connected to the cables and stimulation equipment, but no stimulation was delivered during the treatment-paired training sessions.

### ICMS Motor Mapping

Within 24 h after the final behavioral session, animals were anesthetized with ketamine/xylazine (80/10 mg/kg, i.p.) and, in VNS-treated rats, cuff function validated by evoking the Hering–Breuer reflex (Paintal, 1973; Bucksot et al., 2020). ICMS mapping was then performed as previously described (Porter et al., 2012; Hulsey et al., 2016, 2019; Morrison et al., 2019; Tseng et al., 2020). Briefly, a large craniotomy was made to expose the left motor cortex (ca. +4.0 to −3.0 mm anterior/posterior and ca. +0.2 to +5 mm lateral to bregma). A low impedance tungsten electrode (300–500 kΩ; FHC, Bowdoin, ME, United States) was placed at randomly chosen sites within a grid (0.5 mm spacing) over M1. The electrode was lowered to a depth of 1.8 mm ventral to the pial surface and high-frequency microstimulation was delivered using 40 ms pulse trains made up of 200 μs monophasic cathodal pulses at 300 Hz. Stimulation amplitude was increased from 20 to 200 μA until a motor movement was first evoked. If no motor movement was evoked, sites at −1.6 and −2.0 mm were additionally tested. If no movement was evoked at any depth, the site was marked as non-responsive. Mapping was performed by two experimenters to reduce procedural bias: the first experimenter placed the stimulation electrode, and the second experimenter, blinded to both the treatment condition and the electrode location, determined the threshold amplitude and evoked movement. Across all animals and responsive sites, the median threshold stimulation amplitude was 100 μA (IQR = 70–160 μA). Threshold-evoked motor movements were classified as proximal or distal forelimb, anterior body (vibrissa, jaw, and neck), or posterior body (trunk, hindlimb, and tail) movements.

### Immunohistochemistry and Lesion Quantification

Immediately following ICMS mapping, rats were deeply anesthetized with sodium pentobarbital (150 mg/kg, i.p.) and transcardially perfused with cold (4°C) phosphate buffered saline (PBS) followed by 4% paraformaldehyde in PBS. Brains were removed and stored in 4% paraformaldehyde solution for 4–12 h, then transferred to 30% sucrose in PBS for cryoprotection.

To validate the dopaminergic lesions, coronal slices (30 μm) were made through the motor cortex using a cryostat and immunolabeled for tyrosine hydroxylase (TH). For each rat, three slices containing the forelimb region of M1 were stained and imaged for fiber density analysis. M1 sections were taken every 200 μm from ca. 1.2 to 0.8 mm anterior to bregma, washed in PBS, then permeabilized with 0.5% Triton X-100 in PBS for 30 min. Slices were again washed with PBS, and blocked in a 2.0% BSA in PBS solution for 1 h. Sections were incubated overnight at 4°C in primary antibody (chicken anti-TH, 1:1000 dilution, Abcam #ab76442, RRID:AB_1524535), rinsed with PBS, then incubated in secondary antibody for 1 h at room temperature (anti-chicken IgY conjugated to Alexa Fluor 555, 1:1000 dilution, Abcam #ab150170, RRID:AB_2864276). Slices were then rinsed with PBS and mounted on slides with a DAPI containing mounting medium (SouthernBiotech #0100-20).

**FIGURE 2:**
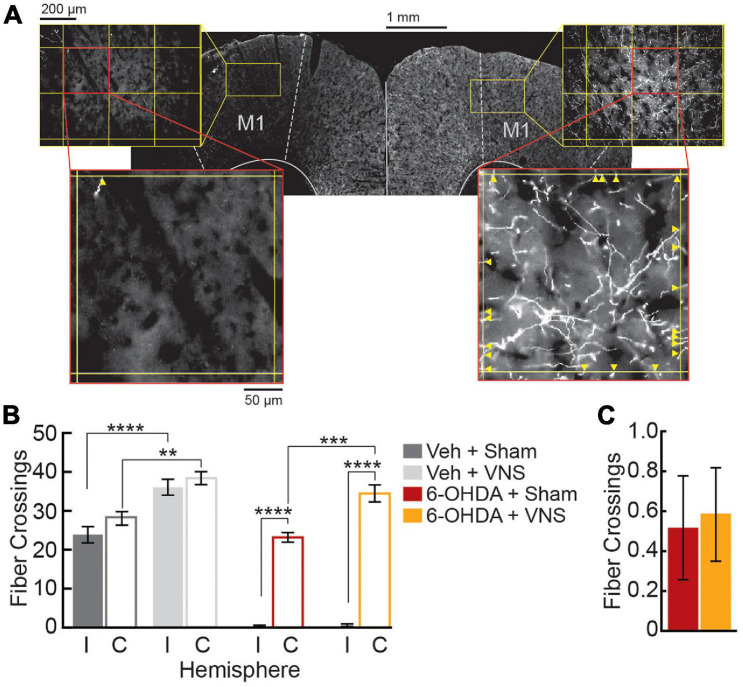
M1 6-OHDA lesions result in severe depletion of TH+ fibers. **(A)** Representative M1 slice from a 6-OHDA infused subject, stained for TH. Fiber crossings (yellow arrowheads) of an overlaid grid (250 μm spacing) were counted within M1 ROIs to quantify TH+ fiber innervation in ipsilesional (left) and contralesional (right) hemispheres. **(B)** Fiber crossings were significantly reduced in ipsilesional (I) compared to contralesional (C) hemispheres following 6-OHDA infusions. VNS treatment increased TH+ fiber innervation within intact M1 hemispheres; this was true in contralateral M1 in both 6-OHDA and vehicle-infused groups, as well as in saline-infused M1. **p < 0.01; ***p < 0.001; ****p < 0.0001, Tukey *post hoc* tests. **(C)** VNS treatment did not alter TH+ fiber counts in 6-OHDA infused M1.

TH+ fiber counts were quantified in M1 bilaterally. For each slice, 600 × 900 μm regions of interest (ROIs) were imaged at 20× magnification using an Olympus BX51 fluorescent microscope (Tokyo, Japan, RRID:SCR_018949). Two ROIs were imaged per slice, one in M1 ipsilateral to the 6-OHDA injection site, and one in contralateral M1. Each ROI was centered 1.0 mm from the midline and 0.7 mm below the pial surface. To quantify TH+ fiber innervation in each ROI, a grid was overlaid on each image (250 μm line spacing) (Figure 2A). Two graders blinded to the treatment condition of each slice counted each fiber crossing of a grid line. For each hemisphere, fiber crossings were averaged across the three slices to obtain an estimate of ipsilesional and contralesional M1 fiber innervation for each subject.

### Statistical Analyses

Statistical analyses of fiber counts, behavioral data, and cortical motor mapping data were performed in Matlab, and results were plotted using Matlab or GraphPad Prism 8. All descriptive statistics are reported as mean ± SEM.

To compare TH+ fiber counts in M1, we performed a three-way ANOVA on lesion condition, stimulation condition, and cortical hemisphere. These were followed by Tukey *post hoc* tests to compare the ipsilesional and contralesional fiber counts within each treatment group, and to compare the effect of VNS vs. sham treatments on cortical TH+ fiber counts in the infused (ipsilesion) and uninfused (contralesion) hemispheres. Results for all comparisons are reported as significant if *p* < 0.05.

We used two-way ANOVAs to determine whether treatment-dependent differences in behavioral performance existed within each behavioral training epoch. Behavioral parameters examined included time to reach criterial performance, trials per session, percent correct performance, lever pressing speed, trial duration, and total VNS or sham stimulations delivered during treatment. Session-wise parameters (trials per session and percent correct) were averaged across the 10 sessions in each training epoch (acquisition, recovery, and stimulation) to obtain a single estimate for each time period for each subject, prior to performing group-wise analyses. For trial-wise parameters (lever pressing speed and trial duration), means were first computed across all correct (“hit”) trials in each session, then averaged across sessions for each subject, prior to group-wise analyses. For each hit trial, trial duration was computed as the time from trial initiation to hit detection, and pressing speed was calculated as the maximum value of the derivative of the lever position within this same trial period.

To compare treatment-related changes in behavior across training epochs, data for each rat was divided by its performance in the immediately preceding epoch, and a two-way ANOVA was performed on the normalized data to determine whether any significant changes resulted from VNS or 6-OHDA treatments. Within-group analyses comparing behavioral performance across training epochs were also performed using paired *t*-tests. All behavioral comparisons are reported as significant if *p* < 0.05.

Total motor cortical map area, along with map areas of PFL, DFL, anterior, and posterior body representations were analyzed using two-way ANOVAs, followed by Tukey *post hoc* comparisons. For comparisons across treatment groups of total motor map size, results are reported as significant for *p* < 0.05. As cortical areas for each map subregion are not independent, all statistical comparisons of individual subregion sizes are reported as significant for a Bonferroni adjusted *p* < 0.0125.

## Results

Twenty-four rats were trained on the skilled reaching lever press task prior to VNS implant surgery and intracortical 6-OHDA (or saline) infusions (Figures 1A–C). No differences in behavioral performance were observed across treatment groups during initial task acquisition (Figure 1D and Table 1). Following task acquisition, rats were randomly assigned to one of the four main treatment groups (veh| sham: *n* = 6; veh| VNS: *n* = 6; 6-OHDA| sham: *n* = 6; 6-OHDA| VNS: *n* = 6).

**TABLE 1:** Behavioral performance during acquisition did not differ across treatment groups.

	**Vehicle (no lesion)**		**6-OHDA (DA lesion)**		**Two-way ANOVA**		
	**Sham (*n* = 6)**	**VNS (*n* = 6)**	**Sham (*n* = 6)**	**VNS (*n* = 6)**			
	**Group Mean (SEM)**				**p_6OHDA_ (F_6OHDA_)**	**p_VNS_ (F_VNS_)**	**p_int_ (F_int_)**
Sessions to criteria	13.0 (1.9)	20.0 (3.9)	20.8 (8.8)	15.2 (4.4)	0.782 (0.08)	0.902 (0.02)	0.250 (1.40)
Trials per session	193.8 (20.1)	199.9 (15.8)	199.9 (11.1)	217.4 (6.7)	0.422 (0.67)	0.419 (0.68)	0.697 (0.16)
Percent correct	75.9 (2.6)	78.2 (3.5)	80.3 (3.1)	78.1 (3.4)	0.502 (0.47)	0.979 (0.0)	0.477 (0.52)
Lever pressing speed (degrees/s)	150.3 (15.7)	137.8 (9.2)	141.0 (14.7)	147.0 (10.6)	0.997 (0.0)	0.801 (0.07)	0.479 (0.52)
Trial duration (s)	0.474 (0.05)	0.563 (0.05)	0.529 (0.08)	0.529 (0.07)	0.872 (0.03)	0.491 (0.49)	0.485 (0.51)

*Behavioral performance parameters were quantified for each treatment group during the 10 sessions during which the rats first achieved criterial behavioral performance. Two-way ANOVA revealed no significant behavioral effects of future lesion or stimulation group assignment during initial task acquisition.*

### VNS Enhances Estimates of TH+ Neurite Density in Intact, but Not DA-Depleted, Cortex

Once rats achieved criterial behavioral performance, VNS cuff electrodes were implanted, and intracortical infusions of 6-OHDA (or saline) were performed to lesion dopaminergic fibers in M1 contralateral to the trained forelimb. Immunofluorescence analyses confirmed that TH+ fiber counts in M1 were significantly depleted following intracortical 6-OHDA infusions (Figure 2). A three-way ANOVA revealed significant effects of 6-OHDA administration, M1 hemisphere, and VNS treatment on TH+ fiber counts, as well as significant interactions between 6-OHDA and M1 hemisphere, and between lesion group and VNS treatment (TH+ fiber crossings in M1, three-way ANOVA; 6-OHDA vs. veh: *F* = 188.3, *p* < 0.0001; intact vs. lesioned M1: *F* = 163.7, *p* < 0.0001; VNS vs. sham: *F* = 47.3, *p* < 0.0001; lesion × hemisphere interaction: *F* = 103.0, *p* < 0.0001; lesion × stimulation interaction: *F* = 5.18, *p* = 0.028; VNS × hemisphere interaction: *F* = 3.63, *p* = 0.0639). Tukey *post hoc* tests confirmed that intracortical 6-OHDA infusion resulted in a dramatic reduction of TH+ fibers in M1 ipsilateral to the injection site, in both sham- and VNS-treated rats (Figure 2B; ipsilesional vs. contralesional M1 TH+ fiber counts, Tukey *post hoc* tests; veh| sham: *p* = 0.6020; veh| VNS: *p* = 0.9661; 6-OHDA| sham: *p* < 0.0001; 6-OHDA| VNS: *p* < 0.0001).

In DA-depleted hemispheres, VNS treatment had no effect on the number of TH+ fiber crossings observed (Figure 2C; ipsilesional M1 TH+ fiber counts, Tukey *post hoc* test; 6-OHDA| sham vs. 6-OHDA| VNS: *p* > 0.999). In rats that received vehicle infusions, however, VNS was found to significantly increase TH+ fiber crossings in M1 ipsilateral to the injection site (Figure 2B; ipsilesional M1 TH+ fiber counts, Tukey *post hoc* test; veh| sham vs. veh| VNS: *p* = 0.0001). Similar effects of VNS were observed in the intact hemispheres contralateral to the infusion sites: a two-way ANOVA on fiber crossings in contralesional M1 revealed a highly significant effect of VNS, as well as a significant effect of 6-OHDA treatment (contralesional M1 TH+ fiber counts, two-way ANOVA; 6-OHDA vs. veh: *F* = 6.42, *p* = 0.0197; VNS vs. sham: *F* = 38.82, *p* < 0.000; interaction: *F* = 0.07, *p* = 0.798). *Post hoc* comparisons showed no significant differences in fiber counts in the contralesional hemisphere between vehicle and 6-OHDA treated rats (contralesional M1 TH+ fiber counts, Tukey *post hoc* test; veh| sham vs. 6-OHDA| sham: *p* = 0.230, veh| VNS vs. 6-OHDA| VNS: *p* = 0.396), suggesting that if there was an impact of 6-OHDA administration in the contralesional hemispheres, this effect was quite small. Tukey *post hoc* tests did confirm that VNS-treated rats exhibited increased TH+ fiber counts in contralesional M1 compared to sham-treated rats (Figure 2B; contralesional M1 TH+ fiber counts, Tukey *post hoc* test; veh| sham vs. veh| VNS: *p* = 0.002, 6-OHDA| sham vs. 6-OHDA| VNS: *p* = 0.001).

In separate rats, 6-OHDA mediated DA lesions were estimated to fully cover the forelimb motor area, and to extend to more than 75% of the total cortical motor map (Supplementary Figure 1). Taken together, these results demonstrate that 6-OHDA administration resulted in a high level of dopamine denervation within the targeted forelimb region of M1. Further, VNS treatment led to a dramatic increase in TH+ fiber crossings within intact motor cortices in both hemispheres. VNS did not, however, rescue TH+ fiber expression after 6-OHDA mediated DA depletion.

### Cortical DA Depletion Did Not Alter Lever-Press Performance

**FIGURE 3:**
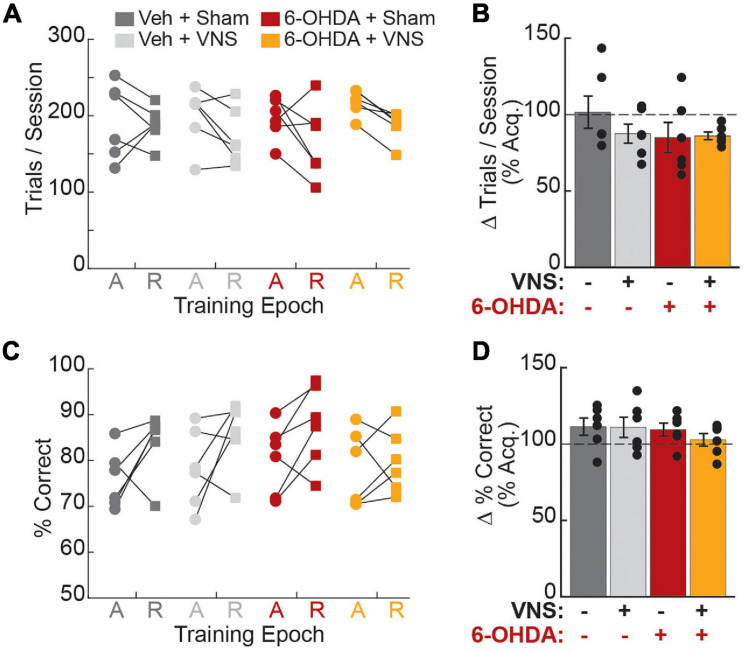
6-OHDA administration did not affect behavioral performance. **(A)** The average trials performed per session did not differ across treatment groups during the acquisition (A, circles) or recovery (R, squares) training epochs. **(B)** Average trials performed was not impacted by 6-OHDA infusions or VNS group assignment. **(C)** The percentage of rewarded trials per session did not differ across treatment groups during acquisition or recovery epochs. **(D)** Percent correct performance was not impacted by 6-OHDA infusions or VNS group assignment. In **(B,D)**, recovery period performance is plotted as a percent of acquisition (Acq.) performance. Legend for all plots is shown in **(A)**.

As cortical DA is critical for motor skill learning, we next examined whether M1 DA depletion impacted task performance in our study. Using two-way ANOVA, we found that neither 6-OHDA infusion, nor VNS group assignment, affected the number of sessions required to re-establish criterial performance after surgery (Table 2). During the six sessions immediately prior to VNS or sham stimulation (recovery period), the average number of trials performed and percent correct performance per session also did not differ across treatment groups (Figure 3 and Table 2). To further assess whether cortical DA depletion resulted in a change in behavioral performance, we normalized each rat’s performance during the pre-stimulation recovery period to their performance during the acquisition period. Two-way ANOVAs revealed no significant change in trials performed or percent correct performance resulting from 6-OHDA infusions or VNS group assignment (Table 2). Paired *t*-tests also confirmed that 6-OHDA treatment did not result in a significant change in these performance parameters following surgery (Supplementary Table 1). As DA depletion could result in movement slowing that might not be captured by success rates, we additionally tested whether lever pressing speeds or total trial durations were altered after surgery. Two-way ANOVAs and paired *t*-tests revealed no main effect of 6-OHDA administration or VNS group assignment on either measure (Table 2, Supplementary Figure 2, and Supplementary Table 1).

**TABLE 2:** Behavioral performance during the recovery (pre-stimulation) period did not differ across treatment groups.

	**Vehicle (no lesion)**		**6-OHDA (DA lesion)**		**Two-way ANOVA**		
	**Sham (*n* = 6)**	**VNS (*n* = 6)**	**Sham (*n* = 6)**	**VNS (*n* = 6)**			
	**Group Mean (SEM)**				**p_6OHDA_ (F_6OHDA_)**	**p_VNS_ (F_VNS_)**	**p_int_ (F_int_)**
Sessions to performance recovery	2.67 (1.7)	2.17 (0.8)	2.17 (0.8)	1.8 (0.4)	0.693 (0.16)	0.693 (0.16)	0.937 (0.01)
Trials per session (raw)	188.0 (9.8)	172.6 (14.9)	166.3 (19.7)	187.3 (8.1)	0.806 (0.06)	0.843 (0.04)	0.206 (1.71)
Trials per session (% of pre-surgery performance)	101.6 (10.6)	87.5 (6.2)	83.5 (9.5)	86.1 (2.5)	0.230 (1.53)	0.477 (0.53)	0.301 (1.13)
Percent correct (raw)	84.0 (2.9)	85.9 (3.1)	87.7 (3.6)	79.9 (2.8)	0.707 (0.15)	0.353 (0.91)	0.129 (2.51)
Percent correct (% of pre-surgery performance)	111.4 (5.6)	111.0 (6.6)	109.6 (4.4)	102.8 (4.1)	0.356 (0.89)	0.501 (0.47)	0.553 (0.36)
Lever pressing speed (degrees/s)	130.6 (12.9)	154.6 (6.3)	162.0 (22.4)	146.8 (11.0)	0.433 (0.64)	0.769 (0.09)	0.198 (1.79)
Lever pressing speed (% of pre-surgery performance)	90.2 (9.2)	113.6 (5.5)	123.5 (15.8)	106.0 (7.0)	0.234 (1.52)	0.778 (0.08)	0.065 (3.87)
Trial duration (s)	0.540 (0.09)	0.483 (0.07)	0.428 (0.06)	0.557 (0.03)	0.801 (0.07)	0.627 (0.24)	0.216 (1.64)
Trial duration (% of pre-surgery performance)	124.9 (32.4)	86.8 (9.9)	80.9 (4.0)	101.8 (11.3)	0.471 (0.54)	0.669 (0.19)	0.150 (2.26)

*Behavioral performance parameters were quantified for each treatment group during the six sessions immediately prior to VNS or sham stimulation delivery. Two-way ANOVA revealed no significant behavioral effects of cortical dopamine depletion or future stimulation treatment during the post-surgery/pre-stimulation period.*

Combined, our behavioral findings demonstrate that cortical DA depletion did not impair behavioral performance on the well-learned skilled reaching lever press task. These results are consistent with published studies indicating that while cortical DA is critical for initial skill acquisition, it is no longer required after stable proficient performance has been achieved (Molina-Luna et al., 2009).

### VNS Treatment Did Not Impact Task Performance

**FIGURE 4:**
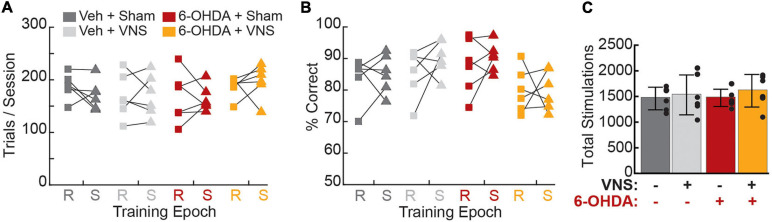
Vagus nerve stimulation administration did not alter behavioral performance. **(A)** Average trials per session did not differ across treatment groups during recovery (R, squares) or stimulation (S, triangles) periods, nor did any group exhibit a significant change in trials performed during VNS or sham stimulation. **(B)** The percentage of rewarded trials per session did not differ across groups during recovery or stimulation epochs, and no group exhibited a significant change in percent correct performance during stimulation sessions. **(C)** Total VNS or sham stimulations delivered, which is identical to the total number of rewarded trials during the final 10 training sessions, did not differ across groups. Legend for all panels is shown in **(A)**.

After criterial performance was re-established post-surgery, rats underwent 10 training-paired VNS (or sham stimulation) sessions. Consistent with previous studies (Porter et al., 2012; Morrison et al., 2019; Tseng et al., 2020), we found that behavioral performance during the stimulation epoch was unaffected by VNS or 6-OHDA treatments (Figure 4, Table 3, and Supplementary Figure 3), though we did observe a significant VNS × 6-OHDA interaction for percent correct performance (Table 3). Subsequent analyses confirmed that there was no treatment-dependent change in performance between the recovery and stimulation training epochs for any behavioral parameter tested (Table 3 and Supplementary Figure 3). Paired *t*-tests further confirmed that VNS did not result in a significant change in trials performed, percent correct, pressing speed, or trial duration between the recovery and stimulation epochs for any group (Supplementary Figure 3 and Supplementary Table 2), demonstrating that stimulation did not significantly impact behavioral performance in either intact or in DA depleted animals.

**TABLE 3:** Behavioral performance during the stimulation period did not differ across treatment groups.

	**Vehicle (no lesion)**		**6-OHDA (DA lesion)**		**Two-way ANOVA**		
	**Sham (*n* = 6)**	**VNS (*n* = 6)**	**Sham (*n* = 6)**	**VNS (*n* = 6)**			
	**Group Mean (SEM)**				**p_6OHDA_ (F_6OHDA_)**	**p_VNS_ (F_VNS_)**	**p_int_ (F_int_)**
Trials per session	171.1 (11.3)	169.6 (16.2)	164.1 (9.9)	197.7 (13.2)	0.421 (0.68)	0.226 (1.56)	0.188 (1.86)
Trials per session (% of pretreatment performance)	92.1 (7.2)	99.6 (9.0)	104.1 (10.6)	106.8 (8.7)	0.296 (1.15)	0.572 (0.33)	0.794 (0.07)
Percent correct	85.2 (2.5)	90.2 (2.3)	90.3 (1.8)	80.0 (2.6)	0.278 (1.24)	0.263 (1.32)	**0.003 (11.25)**
Percent correct (% of pretreatment performance)	101.9 (4.1)	105.8 (5.2)	103.7 (4.1)	101.0 (5.5)	0.751 (0.1)	0.908 (0.01)	0.496 (0.48)
Total stimulations delivered	1461.2 (90.4)	1531.7 (158.5)	1474.3 (68.8)	1612.3 (129.4)	0.693 (0.16)	0.384 (0.79)	0.776 (0.08)
Lever pressing speed (degrees/s)	141.9 (11.7)	150.8 (13.1)	156.6 (22.0)	147.5 (6.6)	0.721 (0.13)	0.994 (0.0)	0.576 (0.33)
Lever pressing speed (% of pretreatment performance)	109.7 (3.0)	96.3 (5.3)	97.3 (3.6)	104.2 (12.9)	0.730 (0.12)	0.681 (0.18)	0.181 (1.94)
Trial duration (s)	0.445 (0.04)	0.437 (0.02)	0.388 (0.06)	0.519 (0.05)	0.803 (0.06)	0.226 (1.58)	0.175 (2.01)
Trial duration (% of pretreatment performance)	86.4 (5.9)	97.3 (10.9)	90.0 (8.0)	96.5 (8.6)	0.883 (0.02)	0.360 (0.89)	0.816 (0.06)

*Behavioral performance parameters were quantified for each treatment group during the 10 sessions in which VNS, or sham stimulation, was paired with correct lever press performance. Two-way ANOVA revealed no significant behavioral effects of VNS or dopamine depletion during the stimulation period. Nor were any significant changes in behavioral performance observed between the recovery (pre-stimulation) and stimulation periods.*

### VNS Induced Motor Map Plasticity Is Not Dependent on Cortical Dopamine

**FIGURE 5:**
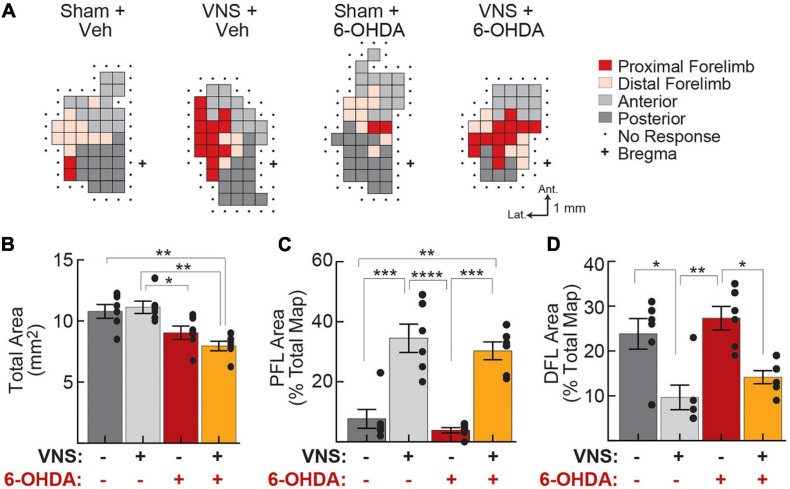
Vagus nerve stimulation drives cortical reorganization even after DA depletion. **(A)** Representative motor maps from each treatment group. Threshold-evoked movements were determined for each site on a grid (0.5 mm spacing) overlying left motor cortex, and sites were classified as representing proximal forelimb (PFL), distal forelimb (DFL), anterior body (jaw, vibrissa, and neck), or posterior body (trunk, hindlimb, and tail) movements. **(B)** Total motor map area was significantly reduced following M1 DA depletion. **(C)** PFL map representations increased as a result of VNS administration, in both vehicle-treated and 6-OHDA infused rats. **(D)** DFL map representations decreased following VNS, in both vehicle- and 6-OHDA infused rats. In **(B–D)**, *p < 0.05; **p < 0.01; ***p < 0.001; ****p < 0.0001, Tukey *post hoc* tests.

Somatotopic cortical motor maps were obtained within 24 h of the final VNS (or sham) treatment sessions (Figure 5A and Supplementary Figure 4). A two-way ANOVA revealed that total M1 map size was significantly impacted by DA depletion, but not by VNS treatment (Figure 5B and Table 4). *Post hoc* comparisons (Supplementary Table 3) revealed that for VNS-treated rats, 6-OHDA infusions resulted in significantly smaller cortical motor maps compared to vehicle infusions groups (total map area, Tukey *post hoc* test; veh| VNS vs. 6-OHDA| VNS: *p* = 0.002). In sham-treated rats, a similar but non-significant trend was observed (total map area, Tukey *post hoc* test; veh| sham vs. 6-OHDA| sham: *p* = 0.10).

**TABLE 4:** Training-paired VNS enhances the task-relevant proximal forelimb (PFL) representation in M1, even after cortical dopamine depletion.

	**Vehicle (no lesion)**		**6-OHDA (DA lesion)**		**Two-way ANOVA**		
	**Sham (*n* = 6)**	**VNS (*n* = 6)**	**Sham (*n* = 6)**	**VNS (*n* = 6)**			
	**Group Mean (SEM)**				**p_6OHDA_ (F_6OHDA_)**	**p_VNS_ (F_VNS_)**	**p_int_ (F_int_)**
Total map area (mm^2^)	10.79 (0.6)	11.13 (0.5)	9.04 (0.6)	7.96 (0.4)	**0.0001** (23.30)	0.470 (0.54)	0.180 (1.93)
Proximal forelimb area (mm^2^)	0.79 (0.3)	3.88 (0.6)	0.33 (0.1)	2.42 (0.3)	0.013 (7.38)	**<0.0001** (53.61)	0.172 (2.01)
Normalized proximal forelimb area	7.59 (3.0)	34.58 (4.7)	3.67 (0.9)	30.45 (3.1)	0.228 (1.55)	**<0.0001** (68.97)	0.959 (0.00)
Distal forelimb area (mm^2^)	2.58 (0.4)	1.04 (0.3)	2.46 (0.2)	1.25 (0.1)	0.939 (0.01)	**<0.0001** (28.24)	0.704 (0.15)
Normalized distal forelimb area	23.86 (3.5)	9.52 (2.7)	27.60 (2.6)	14.15 (1.5)	0.133 (2.28)	**<0.0001** (27.06)	0.870 (0.03)
Anterior movement representation (mm^2^)	3.67 (0.5)	3.50 (0.2)	3.63 (0.7)	2.63 (0.2)	0.318 (1.05)	0.207 (1.70)	0.363 (0.87)
Normalized anterior movement representation	33.27 (3.1)	31.68 (2.4)	39.26 (6.3)	33.16 (2.4)	0.348 (0.93)	0.334 (0.98)	0.567 (0.34)
Posterior movement representation (mm^2^)	3.75 (0.2)	2.71 (0.5)	2.63 (0.7)	1.79 (0.3)	0.042 (4.73)	0.060 (3.99)	0.827 (0.05)
Normalized posterior movement representation	35.29 (2.8)	24.21 (4.1)	29.48 (7.6)	22.24 (3.4)	0.432 (0.64)	0.073 (3.67)	0.696 (0.16)

*Intracortical microstimulation motor mapping was used to create functional somatotopic maps of motor cortex for each subject. Cortical dopamine depletion following 6-OHDA infusions reduced the total motor map area in M1. However, in both vehicle-infused and 6-OHDA infused subjects, VNS treatment significantly expanded PFL map representations and simultaneously decreased distal forelimb representations. Significant two-way ANOVA results for total map area are presented in bold for *p* < 0.05; individual subregions are similarly denoted as significant for Bonferroni-adjusted *p* < 0.0125.*

To account for differences in total map size across animal subjects, for each rat, we computed the percentage of the motor map area composed of PFL, DFL, anterior, and posterior body representations (Figures 5C,D, Table 4, and Supplementary Table 3). Consistent with published literature, we found that VNS paired with correct lever performance enhanced the representation of task-relevant PFL musculature within M1. VNS treatment simultaneously reduced DFL representations but had no significant effect on anterior or posterior body representations. Similar results were obtained when raw areas were considered (Table 4, Supplementary Figure 5 and Supplementary Table 3).

To our surprise, cortical DA depletion did not impact the ability of VNS to drive motor map reorganization (Figures 5C,D and Table 4). Two-way ANOVAs revealed a significant effect of VNS on PFL and DFL representations in M1, but no effect of 6-OHDA; nor was any interaction between VNS and DA depletion observed (Table 4, Supplementary Figure 5, and Supplementary Table 3). Anterior and posterior body representations were also unaffected by 6-OHDA treatments. In a small group of rats (*n* = 3) we performed pharmacological disruption of cortical DA signaling using intra-M1 infusions of the D1 and D2 antagonists SCH 23390 and raclopride. The results of these control experiments were similar to those obtained with 6-OHDA mediated DA depletion (Supplementary Figure 5 and Supplementary Table 4).

Taken together, our data demonstrate that VNS-driven motor map reorganization is unchanged after M1 DA depletion, indicating that cortical dopaminergic innervation is not required for this form of neuroplasticity to occur.

## Discussion

In the current study, we asked whether DA is required for VNS-driven motor cortical plasticity, which is thought to underlie successful rehabilitation after neural injury. Our experiments resulted in two unexpected and novel findings. First, VNS dramatically increased TH+ fiber crossings in intact M1 but failed to rescue catecholaminergic innervation after 6-OHDA mediated cortical DA depletion. Second, even after M1 DA depletion, training-paired VNS significantly increased the task relevant PFL representation within the cortical motor map. Taken together, these results suggest that although VNS may enhance catecholaminergic signaling within the neocortex, cortical DA itself is not necessary for VNS-induced cortical plasticity.

We observed a near-complete loss of TH+ fibers in M1 after 6-OHDA administration. TH+ fibers were eliminated over a large cortical volume, strongly suggesting that our protocol dramatically reduced DA signaling within the forelimb region most likely to be impacted by training-paired VNS. Consistent with prior studies (Hosp et al., 2009), we observed a decrease in cortical map area after disruption of cortical DA signaling, both in 6-OHDA treated rats and in those that received intracortical infusions of DA antagonists, providing further functional evidence that our lesions resulted in significant DA depletion within M1. On the other hand, the near absence of TH+ fibers within M1 after 6-OHDA infusions might suggest that our lesions also depleted noradrenergic fibers despite co-administration of desipramine. However, selective neurotoxic lesioning of noradrenergic fibers within M1 has been previously shown to block VNS-induced map plasticity (Hulsey et al., 2019), suggesting that NE depletion cannot explain our results in the present study. Rather, our finding that VNS continues to induce motor map reorganization after intracortical administration of 6-OHDA suggests that dopaminergic signaling within the motor cortex is not required for this form of cortical plasticity to occur.

We were surprised to find that VNS increased TH+ fiber counts in intact motor cortices. It is not clear whether this increase in TH staining represents the growth of new axonal fibers within M1, or simply an increase in TH expression within existing catecholaminergic neurons, but either possibility suggests that cortical catecholaminergic innervation is likely enhanced following VNS treatment. It is also not clear whether VNS increased both dopaminergic and noradrenergic fibers in M1, or whether one population was disproportionately affected. It is worth noting that we did not observe a significant increase in TH+ fiber density in DA depleted hemispheres, suggesting that VNS is unlikely to dramatically enhance noradrenergic staining when DA fibers are not present. Taken together, these findings raise the possibility that after VNS treatment a significant proportion of new TH+ cortical axons are indeed dopaminergic.

Our finding that VNS-driven cortical plasticity does not depend on intact dopaminergic innervation of the neocortex suggests that alternative neuromodulatory mechanisms are sufficient to promote this specific form cortical plasticity in healthy animals. Notably, however, in intact cortices, we find that VNS paired with motor training enhanced catecholaminergic innervation. New motor learning has been shown to depend on intact cortical dopaminergic signaling (Molina-Luna et al., 2009; Hosp et al., 2011; Rioult-Pedotti et al., 2015). Levodopa administration has also been reported to have a beneficial effect on motor recovery following stroke (Floel et al., 2005; Rösser et al., 2008; Ruscher et al., 2012). Thus, though cortical dopamine was not necessary for plasticity induction in our study, our results suggest that VNS may nonetheless provide additional neuroplastic benefits during initial skill learning or injury recovery by enhancing cortical dopaminergic tone. Motor learning is also well-known to depend on dopaminergic signaling within the basal ganglia (Graybiel, 2005; Wickens, 2009), and additional research is needed to clarify the role of these subcortical circuits following injury recovery and during VNS-enhanced rehabilitation.

Motor skill and sensory discrimination learning are generally accompanied by reorganization of cortical maps *in vivo* (Recanzone et al., 1992; Conner et al., 2003, 2010; Doyon and Benali, 2005; Reed et al., 2011). However, over time, once tasks become well-learned, cortical maps revert to a macro structure similar to that found in naïve animals, without an accompanying decrement in behavioral performance (Molina-Luna et al., 2008; Porter et al., 2012; Reed et al., 2011). These findings suggest that while map reorganization likely reflects plasticity processes that are relevant for initial learning, maintenance of proficient task performance does not depend on map expansion *per se*. Similarly, dopaminergic innervation of the motor cortex is critical for learning new motor skills (Molina-Luna et al., 2009; Hosp et al., 2011; Rioult-Pedotti et al., 2015), but is not necessary for maintenance of good performance after a task becomes well-learned (Hosp et al., 2011). Our results are consistent with this published work, as well as several studies demonstrating that training-paired VNS does not significantly impact proficient lever press performance in healthy rats (Porter et al., 2012; Hulsey et al., 2016, 2019; Morrison et al., 2019). In the current study, we similarly find that cortical dopamine depletion did not impair the performance of a previously well-learned motor task, nor did VNS-induced map reorganization during the late stage of skilled performance alter task execution.

Preclinical studies have shown, however, that expansion of the forelimb area of the cortical motor map is correlated with recovery of limb function after injuries such as stroke (Hosp and Luft, 2011; Meyers et al., 2018; Joy and Carmichael, 2020). Disruption of synaptic function within these expanded map regions after recovery leads to a reinstatement of motor deficits in stroke lesioned rats (Okabe et al., 2016), suggesting that motor cortical plasticity is crucial for functional recovery to occur. Recently published work has also provided evidence that cortical plasticity underlies VNS efficacy (Meyers et al., 2018, 2019). Our finding that dopaminergic innervation is not necessary for VNS-driven cortical plasticity thus raises the intriguing possibility that VNS-enhanced stroke recovery may depend on a unique set of cortical plasticity mechanisms compared to other forms of motor learning.

## Data Availability Statement

The raw data supporting the conclusions of this article will be made available by the authors, without undue reservation.

## Ethics Statement

The animal study was reviewed and approved by the Institutional Animal Care and Use Committee at the University of Texas at Dallas.

## Author Contributions

JB and CT conceived the experiments, analyzed the data, and wrote the manuscript. JB, CS, UA, and KG performed the experiments. All authors contributed to the article and approved the submitted version.

## Conflict of Interest

The authors declare that the research was conducted in the absence of any commercial or financial relationships that could be construed as a potential conflict of interest.

## Publisher’s Note

All claims expressed in this article are solely those of the authors and do not necessarily represent those of their affiliated organizations, or those of the publisher, the editors and the reviewers. Any product that may be evaluated in this article, or claim that may be made by its manufacturer, is not guaranteed or endorsed by the publisher.
