# Supplementary material for: Vagus Nerve Stimulation Induced Motor Map Plasticity Does Not Require Cortical Dopamine
Source: Front Neurosci. 2021 Aug 23;15:693140. doi: 10.3389/fnins.2021.693140 (PMC8420970; doi:10.3389/fnins.2021.693140)
Supplement: Supplementary file 1 [file Data_Sheet_1.docx]

Supplementary Material

# Supplementary Methods

Estimation of 6-OHDA Lesion Spread. To estimate the total cortical area impacted by 6-OHDA infusions, two naïve rats received intracortical infusions of 6-OHDA identical to those in the main study (Fig. S1). Two weeks after infusions, rats were sacrificed, and coronal slices (30 μm) made through the extent of motor cortex (AP: +4.0 to -2.0). Every other slice was stained for TH, and imaged at 10x. Images were loaded into ImageJ (RRID: SCR_003070) and converted to greyscale. Fiber presence was detected through application of upper and lower brightness thresholds (upper: 57; lower: 33), with pixel values above the upper threshold labeled in red (Fig. S1A). Contiguous lesion ROIs were drawn along the boundary using the freehand selection tool. For each image, ROIs were accepted for analysis if detected signal (red pixels) made up less than 1% of the total ROI area. Lesion boundaries then were overlaid on composite cortical motor maps that represented the average maps for the veh|sham and veh|VNS treatment groups (Fig. S1B). To compute the composite motor maps, grid spaces within the map were assigned a specific movement representation (PFL, DFL, anterior or posterior) if the movement response was evoked by ICMS at that location in at least 50% of the subjects in the treatment group. In both subjects, 6-OHDA mediated DA depletion extended to over 70% of the average total cortical map areas, and fully covered the average forelimb areas (PFL + DFL) in both the veh|sham and veh|VNS groups (Fig. S1C).

# Supplementary Figures and Tables

## Supplementary Figures


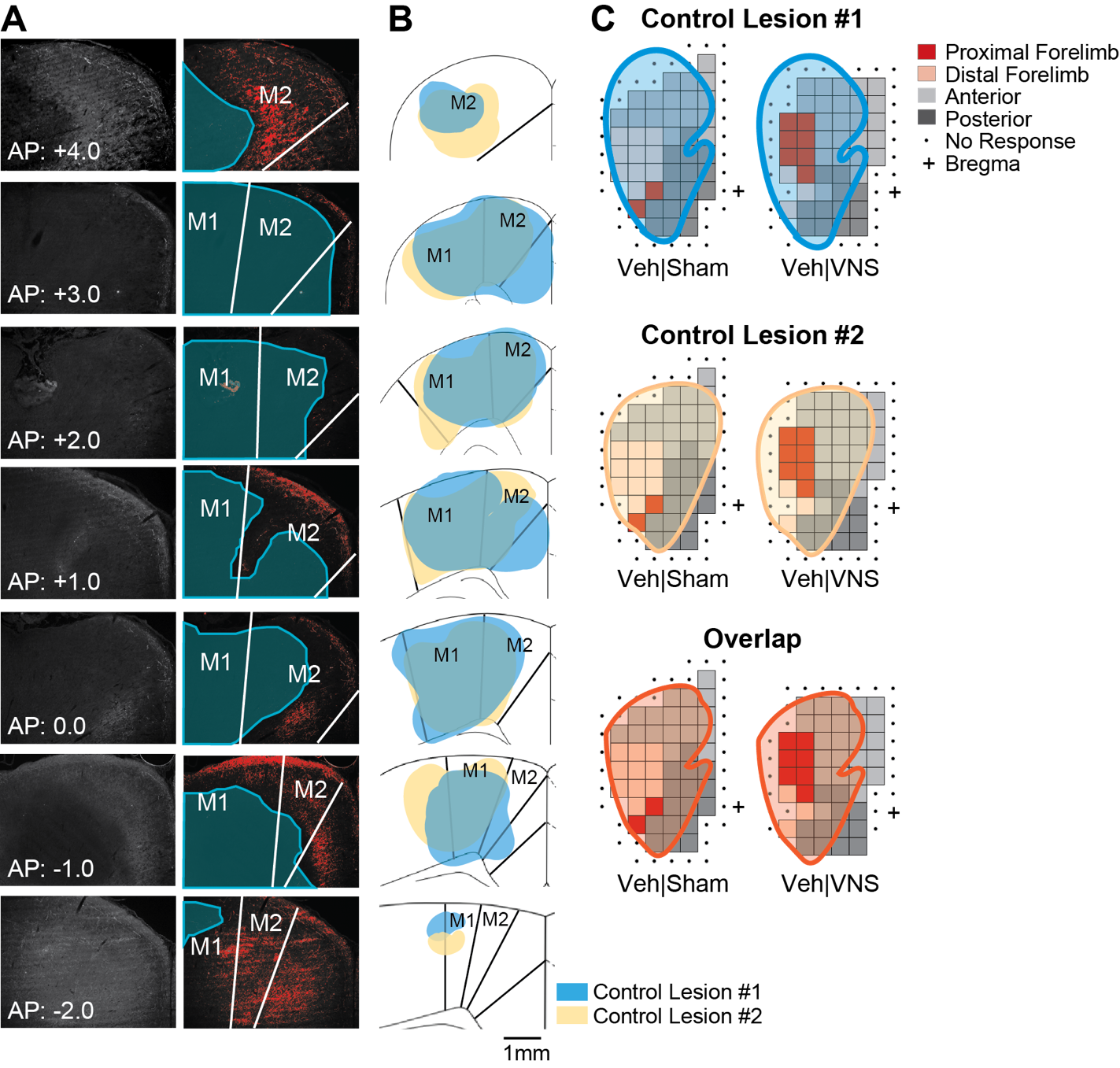


**Figure S1**. 6-OHDA lesions cover the extent of forelimb area in M1. A) Coronal slices through M1 were made following 6-OHDA infusions in 2 naïve rats, were stained for TH, and imaged at 10x (left). Lesion boundaries were hand drawn after applying a consistent brightness threshold to all images (right). B) TH- lesion boundaries for subject 1 (blue) and subject 2 (yellow). C) Lesion boundaries were overlayed on composite motor maps for veh|sham and veh|VNS treatment groups. Lesions in both subjects covered over 70% of the total map area, and fully covered the forelimb region most likely to undergo VNS-driven plasticity.


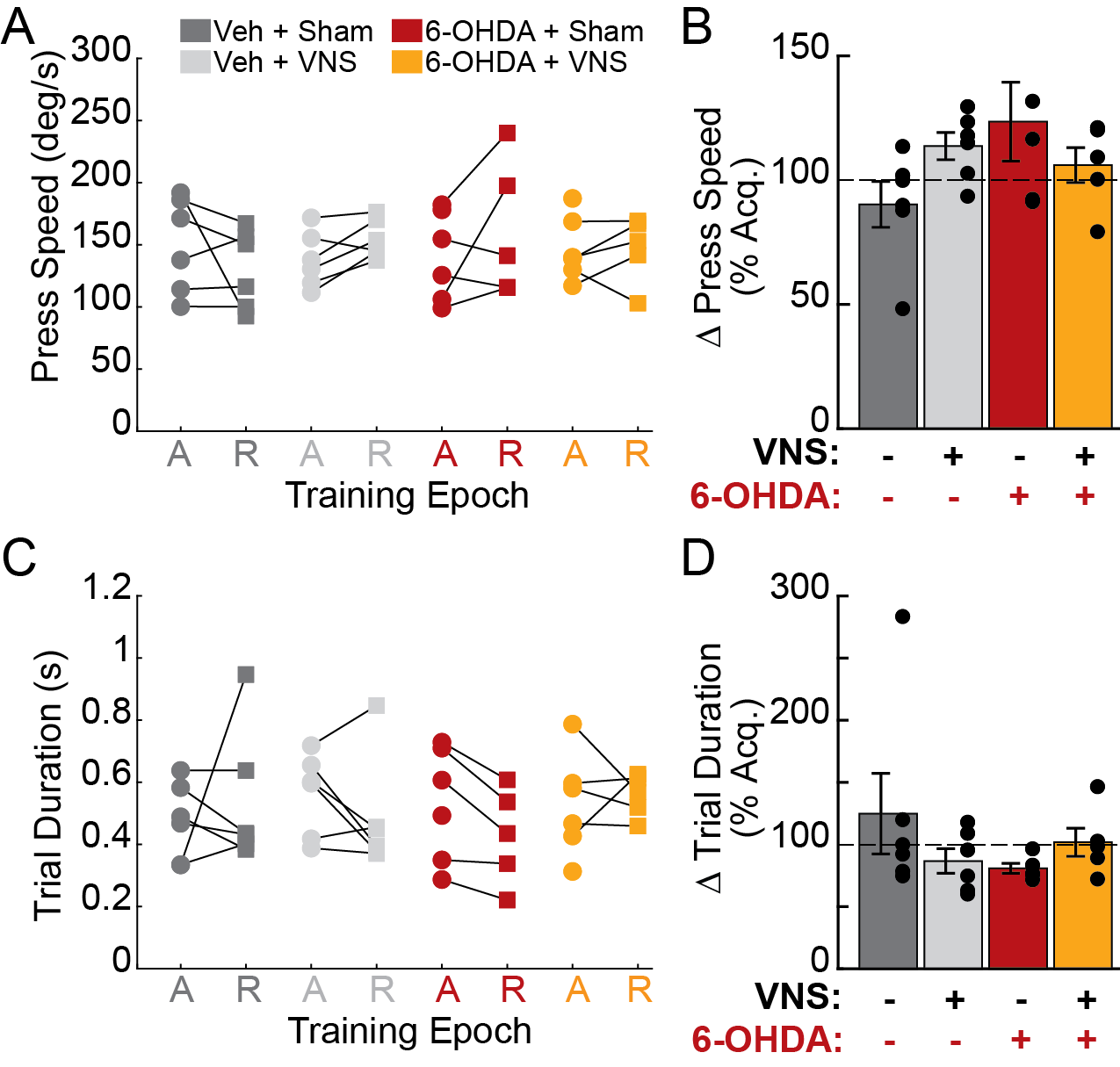


**Figure S2**. Lever pressing speed and trial duration are unaffected by 6-OHDA administration. A-B) Average lever pressing speed does not differ across treatment groups during the 10 sessions in which the rats first met criterial performance (A: Acquisition, circles) or during the 6 sessions immediately prior to delivery of VNS or sham stimulation (R: recovery, squares), nor was pressing speed altered by M1 DA depletion with 6-OHDA. C-D) Trial durations did not differ across groups during acquisition or recovery (C), nor was trial duration impacted by 6-OHDA (D). Color legend for all panels is shown in A.


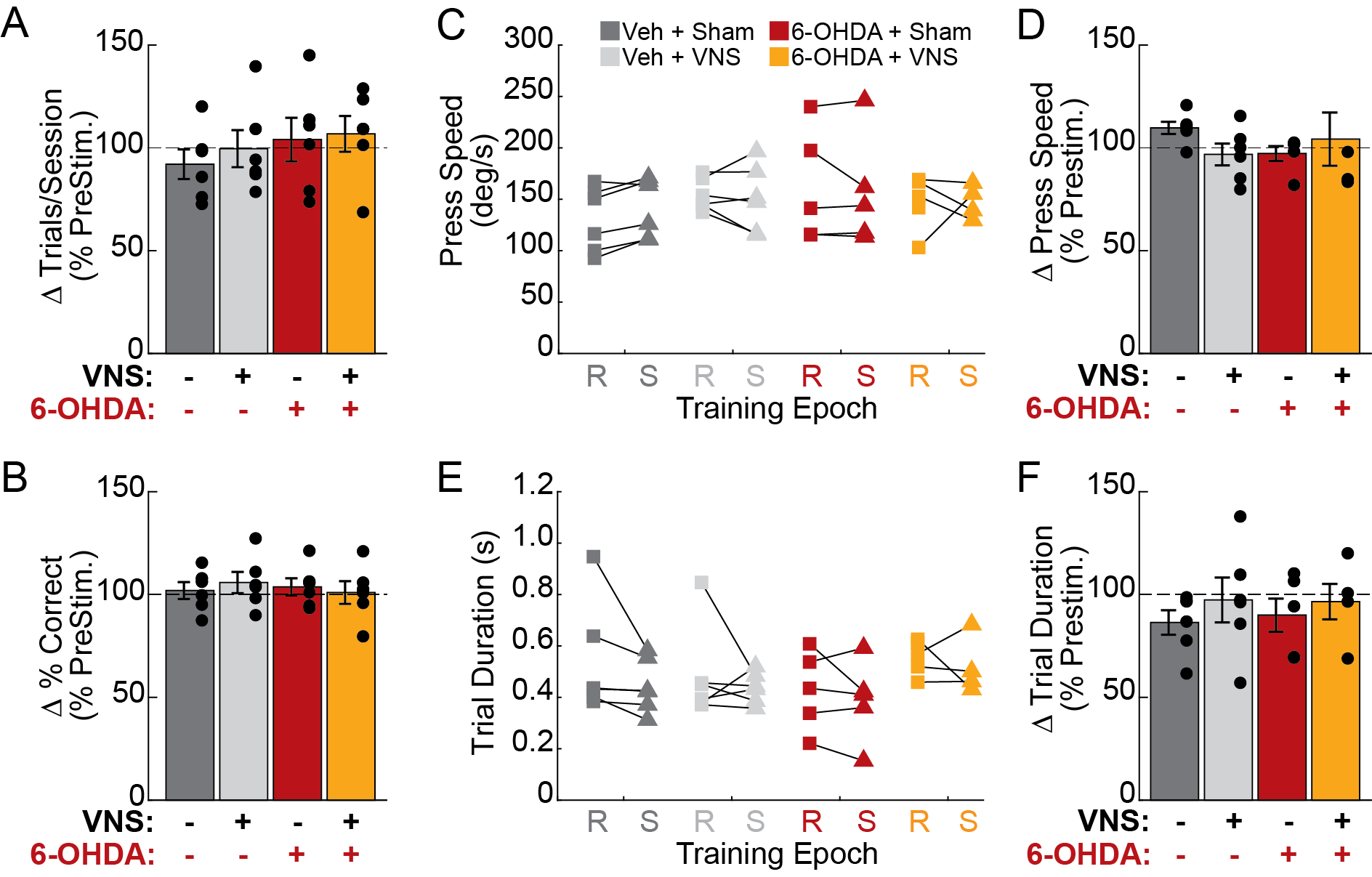


**Figure S3.** VNS treatment does not impact behavioral performance. A) No significant change in the number of trials performed per session was observed during VNS or sham stimulation sessions in any treatment group. B) No change in percent correct performance was observed during VNS or sham stimulation sessions for any group. C-D) Average lever pressing speed did not differ between groups (C) during the 6 sessions immediately prior to VNS or sham stimulation delivery (R: recovery, squares) or during the 10 sessions in which VNS or sham stimulation was delivered (S: stimulation, triangles), nor was pressing speed changed by VNS treatment (D). E-F) Average trial duration does not differ between groups during the recovery or treatment periods (E), nor was trial duration changed by VNS treatment (F). Legend for all panels shown in A.


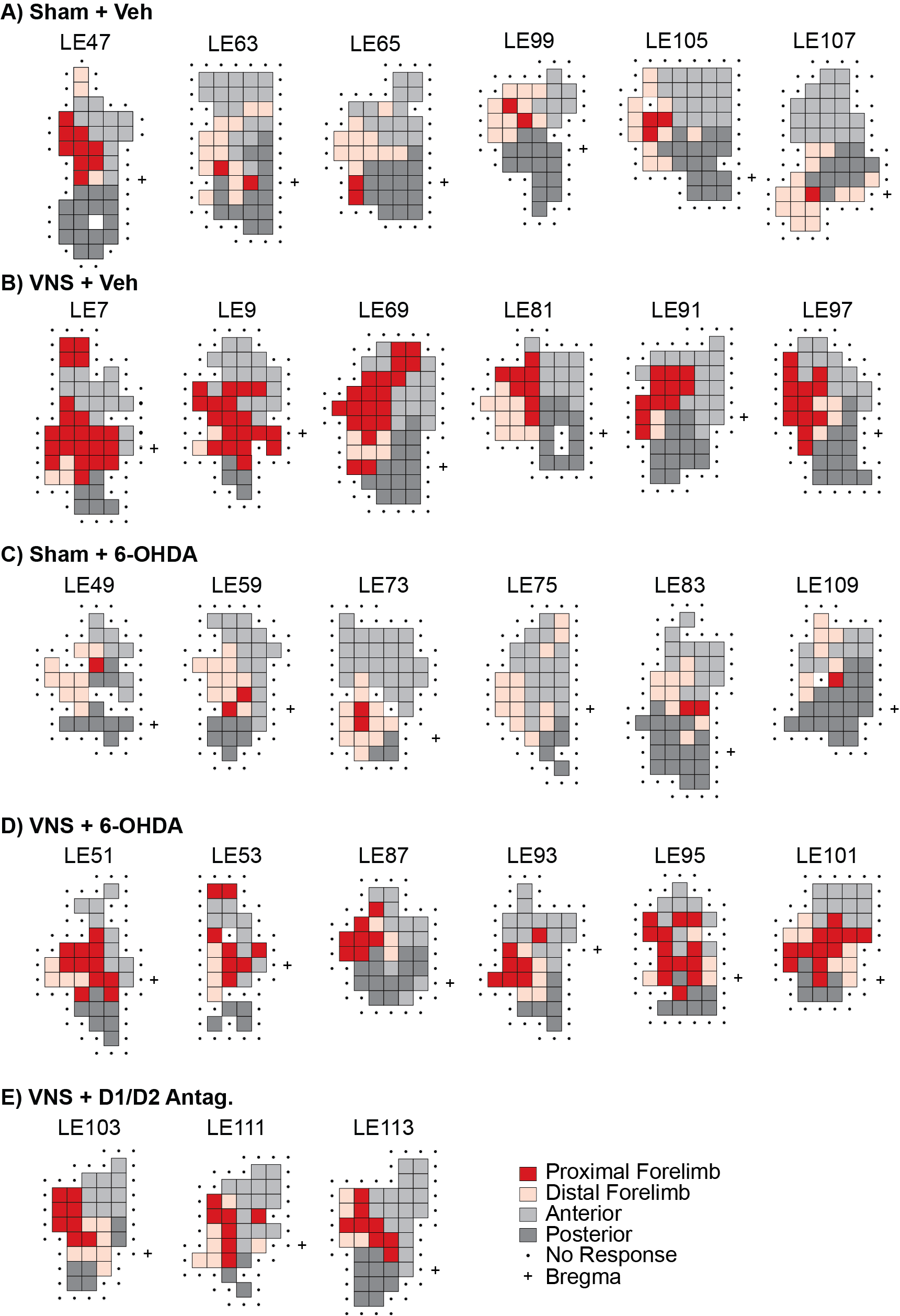


**Figure S4**. ICMS motor maps for all rats included in the study.


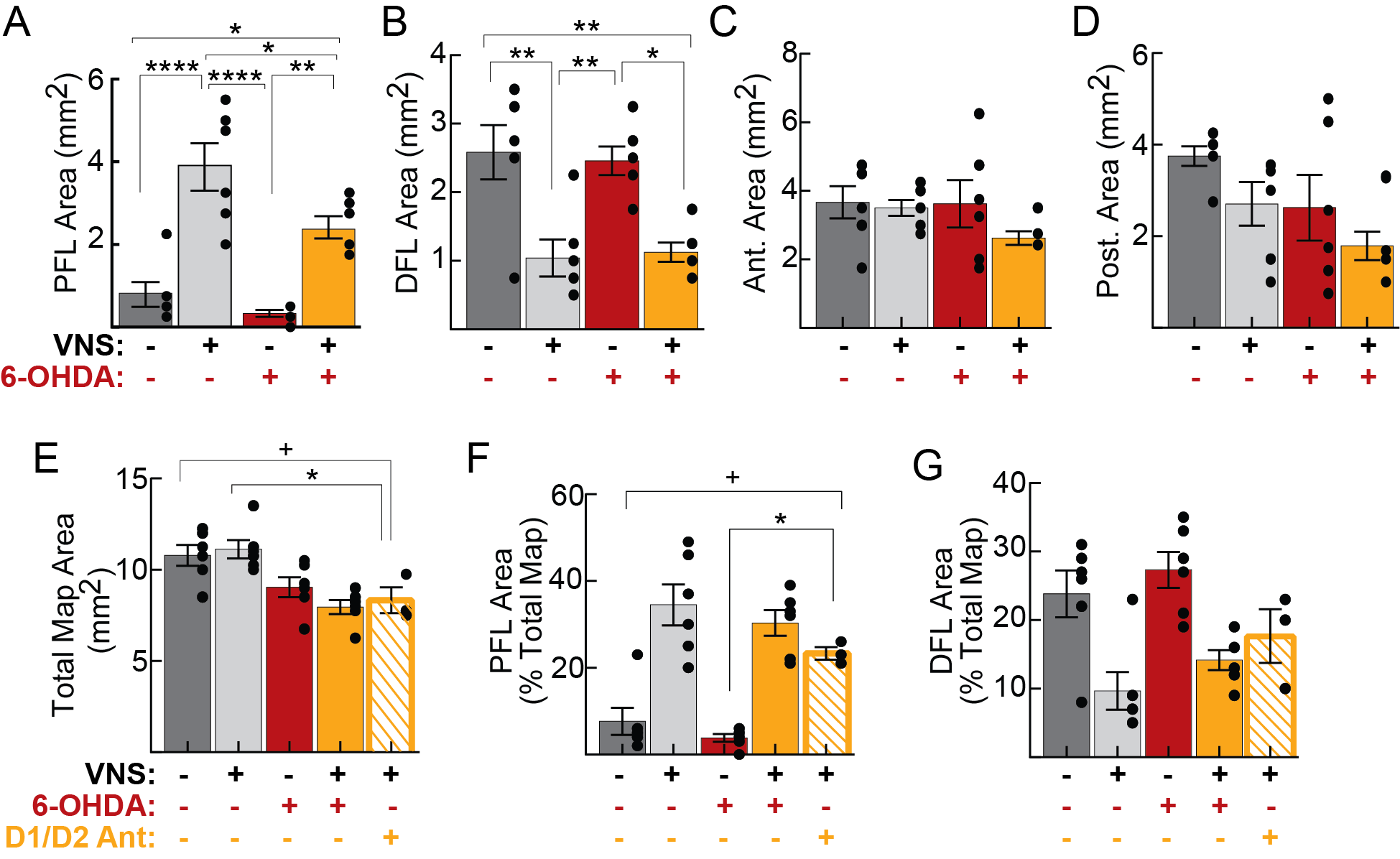


**Figure S5.** VNS-driven motor cortical map reorganization is not blocked by DA depletion. A). Proximal forelimb (PFL) map area expands following VNS treatment, in both vehicle- and 6-OHDA treated rats. B) Distal forelimb (DFL) areas decrease following VNS treatment, in both vehicle- and 6-OHDA infused rats. C-D) Neither 6-OHDA infusions nor VNS treatment significantly impacted anterior (C, vibrissa + jaw + neck) or posterior (D, trunk + hindlimb + tail) representations within the motor map. E-G) Cortical co-infusions of the DA receptor antagonists SCH 23390 and raclopride prior to training-paired VNS treatment sessions produced similar effects as 6-OHDA mediated DA depletion. Intracortical DA antagonist infusions resulted in a significant reduction in total map area (E) and an increase in PFL map representations (F). DFL representations in the small drug group (n = 3) did not differ significantly from any other treatment group. In A-E, +: p < 0.1, *: p < 0.05, **: p < 0.01, ***: p < 0.001, ****: p < 0.0001, Tukey post-hoc comparisons. In F, subregion comparisons were Bonferroni corrected and +: p < 0.025, *: p < 0.0125. For complete post-hoc statistical results, see Table S3 and Table S4.

## Supplementary Tables

**Table S1**. Within-group comparisons of behavior during Acquisition versus Recovery periods

|  | Vehicle | | 6-OHDA | |
| --- | --- | --- | --- | --- |
|  | Sham  (n = 6) | VNS  (n = 6) | Sham  (n = 6) | VNS  (n = 6) |
|  | Paired t-test *p*-value | | | |
| *Percent Correct Performance* | 0.1049 | 0.1563 | 0.0762 | 0.6208 |
| *Trials per Session* | 0.7503 | 0.0926 | 0.1910 | **0.0023** |
| *Lever Press Speed* | 0.2984 | 0.0576 | 0.2320 | 0.4761 |
| *Trial Duration* | 0.5908 | 0.2575 | **0.0252** | 0.8386 |

For each subject and each behavioral parameter of interest, performance during the 10 sessions during which rats first achieved criterial behavioral performance prior to surgery (Acquisition) was compared to the 6 post-surgery sessions immediately prior to VNS or sham stimulation delivery (Recovery). Within group comparisons were performed using paired t-tests and p-values are reported. **Bold** indicates a significant change in performance for p < 0.05. Across all 24 rats, percent correct performance increased and trials per session decreased (percent correct: p = 0.004; trials per session: p = 0.003; paired t-test), consistent with an overall improvement in task performance across all subjects with continued training post-surgery, which we have also observed in previous studies (Tseng et al., 2020). In the current experiment, no decrement in performance was observed after intracortical 6-OHDA infusion.

**Table S2.** Within-group comparisons of behavior during Recovery versus Stimulation periods

|  | Vehicle | | 6-OHDA | |
| --- | --- | --- | --- | --- |
|  | Sham  (n = 6) | VNS  (n = 6) | Sham  (n = 6) | VNS  (n = 6) |
|  | Paired t-test *p*-value | | | |
| *Percent Correct Performance* | 0.7349 | 0.3317 | 0.4676 | 0.9829 |
| *Trials per Session* | 0.2452 | 0.8518 | 0.9015 | 0.5458 |
| *Lever Press Speed* | **0.0194** | 0.6546 | 0.5229 | 0.9808 |
| *Trial Duration* | 0.1502 | 0.5436 | 0.3904 | 0.7323 |

For each subject and each behavioral parameter of interest, performance during the 6 sessions immediately prior to VNS or sham stimulation delivery (Recovery) was compared to the 10 sessions in which VNS or sham stimulation was paired with correct lever performance (Stimulation). Within group comparisons were performed using paired t-tests and p-values are reported. **Bold** indicates a significant change in performance for p < 0.05. No changes in behavior were seen to result from VNS treatment.

**Table S3.** Post hoc comparisons of motor map subregion areas across treatment groups

|  | Sham vs VNS  Comparisons | | Veh vs 6-OHDA  Comparisons | | Cross-treatment  Comparisons | |
| --- | --- | --- | --- | --- | --- | --- |
|  | Vehicle  Groups | 6-OHDA  Groups | Sham  Groups | VNS  Groups | veh\|sham vs.  6-OHDA\|VNS | veh\|VNS vs.  6-OHDA\|sham |
|  | Tukey *post-hoc* test *p*-value | | | | | |
| *Total motor map area (mm^2^)* | 0.9663 | 0.4537 | 0.1035 | **0.0015** | **0.0042** | **0.0412** |
| *PFL area, raw (mm^2^)* | **0.0000** | **0.0024** | 0.7953 | 0.0387 | 0.0190 | **0.0000** |
| *PFL area, normalized*  *(% total map)* | **0.0001** | **0.0001** | 0.8266 | 0.8030 | **0.0004** | **0.0000** |
| *DFL area, raw (mm^2^)* | **0.0034** | **0.0115** | 0.9876 | 0.9962 | **0.0055** | **0.0071** |
| *DFL area, normalized*  *(% total map)* | **0.0057** | **0.0097** | 0.7562 | 0.6184 | 0.0790 | **0.0006** |
| *Anterior area, raw (mm^2^)* | 0.9934 | 0.4115 | 0.9999 | 0.5239 | 0.3768 | 0.9972 |
| *Anterior area, normalized*  *(% total map)* | 0.9914 | 0.6872 | 0.6986 | 0.9930 | 1.000 | 0.5257 |
| *Posterior area, raw (mm^2^)* | 0.4177 | 0.6006 | 0.3524 | 0.5252 | 0.0366 | 0.9993 |
| *Posterior area, normalized*  *(% total map)* | 0.3925 | 0.7194 | 0.8212 | 0.9915 | 0.2584 | 0.8675 |

Somatotopic motor maps were constructed for each subject within 24 hours of the final training-paired VNS or sham stimulation session using ICMS. 2-way ANOVA and post hoc Tukey tests were used to determine the effects of 6-OHDA and VNS treatments on total motor map area, and on the raw and normalized areas of proximal forelimb (PFL), distal forelimb (DFL), anterior body representations (vibrissa + neck + jaw), and posterior body representations (trunk + hindlimb + tail). Table reports p-values for Tukey post-hoc tests for all group-wise comparisons (veh|sham: n = 6; veh|VNS: n = 6; 6-OHDA|sham: n = 6; 6-OHDA|VNS: n = 6). Statistically significant results are denoted in **bold** (p < 0.05 for total map size; Bonferroni corrected p < 0.0125 for all subregion comparisons). 6-OHDA treated rats exhibited smaller total map areas than those that received vehicle infusions (2-way ANOVA on total map area with main effects of lesion and VNS reveals a significant effect of 6-OHDA: p_lesion_ < 0.0001, p_VNS_ = 0.3914, pinteraction = 0.152). VNS significantly increased PFL representations and decreased DFL representations, in both vehicle- and 6-OHDA treated rats.

**Table S4**. Post hoc comparisons of motor map areas for DA antagonist treated rats

| D1/D2 Antagonist Group vs.: | Veh\|Sham  (n = 6) | Veh\|VNS  (n = 6) | 6-OHDA\|Sham  (n = 6) | 6-OHDA\|VNS  (n = 6) |
| --- | --- | --- | --- | --- |
|  | Tukey test *p*-value | | | |
| *Total motor map area (mm^2^)* | *0.0721* | **0.0325** | 0.9267 | 0.9927 |
|  | | | | |
| *PFL area, raw (mm^2^)* | 0.3326 | **0.0215** | *0.0840* | 0.9094 |
| *PFL area, normalized (% total map)* | *0.0567* | 0.2495 | **0.0114** | 0.6675 |
|  | | | | |
| *DFL area, raw (mm^2^)* | 0.1113 | 0.9198 | 0.1844 | 0.9663 |
| *DFL area, normalized (% total map)* | 0.6636 | 0.4260 | 0.2313 | 0.9424 |
|  | | | | |
| *Anterior area, raw (mm^2^)* | 0.9973 | 1.000 | 0.9987 | 0.8248 |
| *Anterior area, normalized (% total map)* | 0.7811 | 0.6432 | 0.9992 | 0.7724 |
|  | | | | |
| *Posterior area, raw (mm^2^)* | *0.0835* | 0.6277 | 0.6904 | 0.9989 |
| *Posterior area, normalized (% total map)* | 0.2666 | 0.9500 | 0.6565 | 0.9886 |

A small group of rats (n = 3) received intracortical infusions of D1 and D2 antagonists 30 minutes prior to each of the 10 training-paired VNS sessions to achieve more temporally restricted disruption of M1 DA signaling during treatment and validate the effects of DA depletion. One-way ANOVA and Tukey post-hoc comparisons were used to compare total motor map areas, as well as proximal forelimb (PFL), distal forelimb (DFL), anterior body representations (vibrissa + neck + jaw), and posterior body representations (trunk + hindlimb + tail) between this pharmacological treatment group and groups in the main study. Table reports p-values for Tukey post-hoc tests for all group-wise comparisons. Statistically significant results are denoted in **bold**, non-significant trends (p < 0.1) are denoted in *italics*. Like 6-OHDA, pharmacological antagonism of DA receptors resulted in a reduction in total map area compared to intact rats. In this group, VNS also significantly increased PFL representations (as a percentage of the total map) compared to those of sham treated rats.
